# Optimal mode of delivery in pregnancy: Individualized predictions using national vital statistics data

**DOI:** 10.1371/journal.pdig.0000166

**Published:** 2022-12-29

**Authors:** Karl W. Schulz, Kelly Gaither, Corwin Zigler, Tomislav Urban, Justin Drake, Radek Bukowski

**Affiliations:** Department of Women’s Health, Dell Medical School, The University of Texas at Austin, Austin, Texas, United States of America; Ben-Gurion University of the Negev, ISRAEL

## Abstract

Child birth via Cesarean section accounts for approximately 32% of all births each year in the United States. A variety of risk factors and complications can lead caregivers and patients to plan for a Cesarean delivery in advance before onset of labor. However, a non-trivial subset of Cesarean sections (∼25%) are unplanned and occur after an initial trial of labor is attempted. Unfortunately, patients who deliver via unplanned Cesarean sections have increased maternal morbidity and mortality rates and higher rates of neonatal intensive care admissions. In an effort to develop models aimed at improving health outcomes in labor and delivery, this work seeks to explore the use of national vital statistics data to quantify the likelihood of an unplanned Cesarean section based on 22 maternal characteristics. Machine learning techniques are used to ascertain influential features, train and evaluate models, and assess accuracy against available test data. Based on cross-validation results from a large training cohort (*n* = 6,530,467 births), the gradient-boosted tree algorithm was identified as the best performer and was evaluated on a large test cohort (*n* = 10,613,877 births) for two prediction scenarios. Area under the receiver operating characteristic curves of 0.77 or higher and recall scores of 0.78 or higher were obtained and the resulting models are well calibrated. Combined with feature importance analysis to explain why certain maternal characteristics lead to a specific prediction in individual patients, the developed analysis pipeline provides additional quantitative information to aid in the decision process on whether to plan for a Cesarean section in advance, a substantially safer option among women at a high risk of unplanned Cesarean delivery during labor.

## Author summary

Child birth via Cesarean section accounts for almost one third of all births each year in the United States. While many of these Cesarean deliveries are planned for before the onset of labor, a subset arise from complications during labor and occur after an initial trial of labor. These unplanned Cesarean sections unfortunately have increased maternal and neonatal morbidity and mortality rates. This work leverages vital statistics data to develop predictive models that quantify the risk of having an unplanned Cesarean section based on 22 maternal characteristics. Multiple derived models were benchmarked against a large testing cohort to identify a clinically practical model that also demonstrated high calibration accuracy. Ultimately, this model can be used to provide a quantitative aid in the decision process on whether to plan for a Cesarean section in advance, a substantially safer option among women at a high risk of encountering an unplanned Cesarean delivery during labor.

## Introduction

Based on data from the Centers for Disease Control and Prevention (CDC), approximately 32% of all live births in the U.S. during the last decade have been performed via Cesarean section (C-section) [1]. Of these C-sections, approximately 25% occur after an initial trial of labor is attempted for vaginal delivery. In total, these unplanned C-sections account for ∼300,000 births each year. Unfortunately, unplanned C-sections are also associated with a two- to threefold increase in maternal morbidity and mortality rates along with poorer fetal outcomes [2–7]. To potentially aid in improving birth-related morbidities and provide additional information for patients and caregivers, this work seeks to develop and test machine-learning models to predict the probability of an unplanned C-section using national vital statistics data published by the CDC. Potential input features for the models are selected based on domain knowledge to account for two prediction scenario timeframes: the first occurring during the 1^st^ trimester (<14 weeks of pregnancy), and the second occurring near the end of pregnancy, where important additional information is available.

The data considered for this analysis is published by the CDC on an annual basis [8] and provides vital statistics information for all U.S. births (approximately 4 million births per year). It is worth noting that the ability to analyze unplanned C-sections as proposed in the current work is facilitated by a change to the U.S. birth certificate that was first introduced in 2003. In particular, the form of the *2003 U.S Standard Certificate of Live Birth* introduced additional checkboxes characterizing labor and the method of delivery including a flag indicating whether labor was attempted or not (recorded in CDC published data as ME_TRIAL). Combined with the method of delivery information, we can thus classify C-sections into labored or non-labored variants for which the labored variant is assumed to be an unplanned C-section.

**Fig 1:**
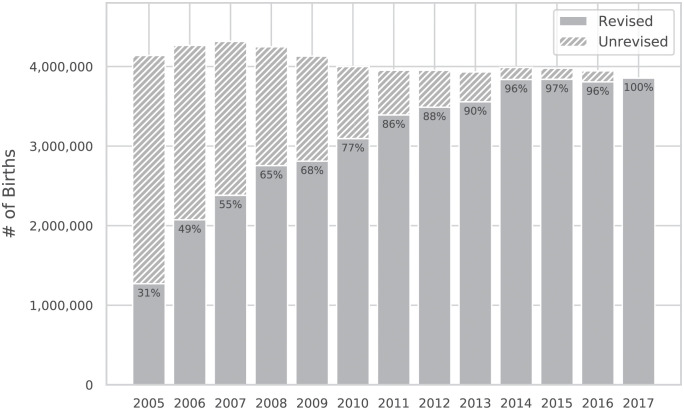
History of the total number of births to U.S. residents per year and the adoption growth of the *2003 Revised Birth Certificate*. The percentage shown each year highlights the fraction of births reported to the CDC that used the revised birth certificate.

Although this birth certificate change was introduced at the national level in 2003, reporting adoption by states slowly increased over a number of years with new statistics first evident in CDC data beginning in 2005. By parsing and analyzing reporting flags in the CDC data, we document the growth in adoption of the revised certificate in Fig 1 which considers all births to U.S residents from 2005 to 2017 (note that 2017 is the most recent year for which the CDC has published period-linked birth information). In 2005, only 30.7% of the births reported had adopted the revised birth certificate; by 2011, this percentage had increased significantly to over 85% and surpassed 95% beginning in 2014. Based on this observed reporting trend and a motivation to include the majority of births in a given year for our analysis, we restrict further discussion in this paper to births reported between 2011-2017 using the revised birth certificate. Note that statistical overviews of the data considered in this analysis are published by the CDC on a year by year basis [9–15].

### Primary outcome of interest

The primary predictive outcome of interest in this work is an unplanned (labored) C-section. We differentiate between labored and non-labored C-sections based on examination of two available CDC variables: ME_TRIAL and RDMETH_REC. The ME_TRIAL variable is simply used to indicate whether labor was attempted or not while the RDMETH_REC variable corresponds to the (revised) delivery method recode and is used to delineate between vaginal or C-section deliveries. Potential values for RDMETH_REC and their classification are as follows [16]:

1 ⇒ Vaginal (excludes vaginal births after previous C-section)
2 ⇒ Vaginal after previous C-section
3 ⇒ Primary C-section
4 ⇒ Repeat C-section
5 ⇒ Vaginal (unknown if previous C-section)
6 ⇒ C-section (unknown if previous C-section)
9 ⇒ Not stated

**Fig 2:**
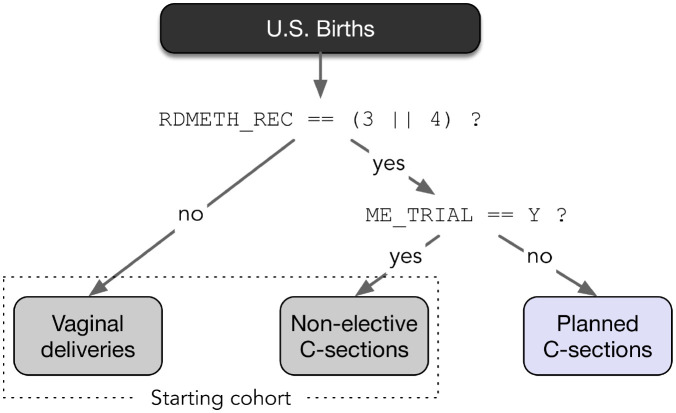
Starting cohort identification of all attempted vaginal deliveries via examination of two variables included in CDC data.

The logic for binning births using these two variables is shown in Fig 2 and defines the starting analysis cohort used for subsequent machine learning analysis. In particular, we consider the group of births comprised of both vaginal deliveries and unplanned C-sections as forming the basis for a supervised machine-learning classification problem. Non-labored (planned) C-sections are excluded from the analysis cohort.

### Modeling variables

A variety of data elements for the mother, father, and baby are included in birth data published by the CDC. For this analysis, we restricted potential modeling variables to a maximum of 22 maternal characteristics and grouped them into two broad sets based on their time of availability: the *t*_*early*_ prediction scenario includes items known during the 1^st^ trimester while the *t*_*term*_ scenario includes additional items known at or near the time of labor and delivery. A full list of the binned variables considered, along with a brief description and their numerical data type is presented in Table 1. Note from Table 1 that two categorical features are included related to the mother’s race and hispanic origin. The encoding for these features is further expanded in Table 2 using CDC-provided classifications.

**Table 1:** Variables from CDC vital statistics data considered for machine-learning models to classify mode of delivery for two prediction scenarios: *t*_*early*_ variables are known during the 1^st^ trimester while *t*_*term*_ variables are known near the time of labor and delivery.

Variable	Variable Description	Feature Type		
		Numerical	Binary	Categorical
variables known at *t*_*early*_				
mager	mother’s age	✓		
lbo	live birth order (recode)	✓		
tbo	total birth order (recode)	✓		
bmi_r	body mass index (recode)	✓		
pwgt_r	pre-pregnancy weight (recode)	✓		
mbrace	mother’s bridged race			✓
umhisp	mother’s hispanic origin			✓
rf_ppterm	previous preterm birth?		✓	
rf_cesar	previous C-sections?		✓	
rf_cesarn	number of previous C-sections	✓		
rf_diab	prepregnancy diabetes		✓	
rf_phyp	prepregnancy hypertension		✓	
cig_0	pre-pregnancy cigarette use	✓		
cig_1	cigarettes usage 1st trimester	✓		
additional variables known at *t*_*term*_				
rf_ghyp	gestational hypertension		✓	
rf_gest	gestational diabetes		✓	
cig_2	cigarette usage 2nd trimester	✓		
cig_3	cigarette usage 3rd trimester	✓		
previs_rec	number of prenatal visits	✓		
combgest	gestational age in weeks	✓		
wtgain_rec	weight gain (recode)	✓		

**Table 2:** Encoding of categorical features for mother’s race and hispanic origin.

mother’s bridged race	
mbrace_1	White
mbrace_2	Black
mbrace_3	American Indian
mbrace_4	Asian Indian
mbrace_5	Chinese
mbrace_6	Filipino
mbrace_7	Japanese
mbrace_8	Korean
mbrace_9	Vietnamese
mbrace_10	Other Asian
mbrace_11	Hawaiian
mbrace_12	Guamanian
mbrace_13	Samoan
mbrace_14	Other Pacific Islander
mbrace_15	More than one race
mother’s hispanic origin	
umhisp_0	Non-Hispanic
umhisp_1	Mexican
umhisp_2	Puerto Rican
umhisp_3	Cuban
umhisp_4	Central or South American
umhisp_5	Other and Unknown Hispanic
umhisp_9	Origin unknown or not stated

## Results

**Fig 3:**
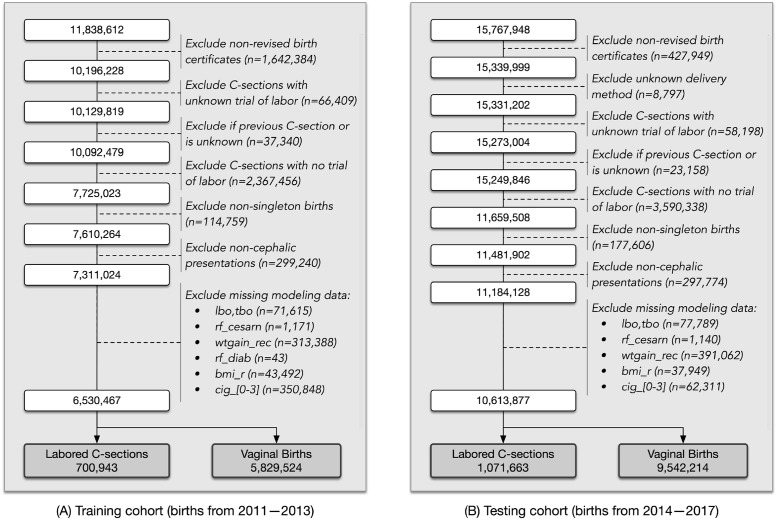
Overview of data filtering applied to CDC data to arrive at analysis cohorts for (A) training and (B) testing of machine-learning classification methods applied to births with a trial of labor.

A comparison study using multiple machine-learning algorithms and parameter feature sets was undertaken to assess whether maternal characteristics that are included in national vital statistics birth data can be used to adequately predict unplanned C-sections. This first analysis was carried out on a training set comprised of births reported to the CDC using revised birth certificates from 2011–2013. The logic of Fig 2 was used to restrict the training cohort to include only births with a known trial of labor. Additional inclusion criteria for the study was to restrict to singleton births, cephalic presentations, and to exclude births with missing values for desired modeling features. A summary of the multi-step data filtering process is shown in Fig 3A indicating assembly of a final training cohort comprised of 6, 530, 467 births. Of the ∼6.5M births that attempted labor, 10.7% of them (700, 943) ultimately delivered via unplanned C-section and form the positive class for evaluation of multiple supervised machine-learning configurations.

Table 3 presents comparative classification performance metrics from multiple ten-fold cross-validation procedures using the training set with three different machine-learning algorithms and varying number of input features from the *t*_*early*_ and *t*_*term*_ prediction scenarios. Execution runtimes obtained on a commodity server are also included. AdaBoost and XGBoost are seen to perform better for this imbalanced classification problem with AUC scores of 76% or better and recall scores of 77% or better (when using a minimum of 10 feature parameters). XGBoost is seen to deliver better (lower) Brier scores in all cases.

**Table 3:** Delivery mode classification performance using varying number of input features from public CDC data and machine-learning models using tenfold cross-validation of the training set (results using variable combinations from the *t*_*early*_ and *t*_*term*_ prediction scenario timeframes). Scoring results from five metrics are presented (mean value ±2*σ*) along with execution runtimes for each cross-validation exercise.

Features	AUC	Accuracy	Recall	F1	Brier	Time (s)
	XGBoost [*t*_*early*_]					
5	0.765 ± 0.009	0.635 ± 0.014	0.789 ± 0.013	0.317 ± 0.007	0.201 ± 0.008	655.4
10	0.769 ± 0.008	0.640 ± 0.019	0.788 ± 0.017	0.319 ± 0.008	0.199 ± 0.011	782.2
15	0.770 ± 0.008	0.640 ± 0.019	0.788 ± 0.017	0.320 ± 0.008	0.198 ± 0.010	769.2
20	0.770 ± 0.008	0.640 ± 0.019	0.788 ± 0.016	0.320 ± 0.008	0.199 ± 0.010	825.5
36	0.770 ± 0.008	0.640 ± 0.019	0.788 ± 0.017	0.320 ± 0.008	0.199 ± 0.010	950.3
	XGBoost [*t*_*term*_]					
5	0.770 ± 0.009	0.657 ± 0.015	0.771 ± 0.014	0.326 ± 0.008	0.198 ± 0.006	662.3
10	0.778 ± 0.007	0.667 ± 0.017	0.769 ± 0.016	0.332 ± 0.009	0.195 ± 0.010	705.7
15	0.780 ± 0.008	0.669 ± 0.019	0.768 ± 0.018	0.333 ± 0.009	0.194 ± 0.011	806.0
20	0.781 ± 0.008	0.668 ± 0.019	0.771 ± 0.016	0.333 ± 0.009	0.194 ± 0.011	843.8
41	0.781 ± 0.008	0.668 ± 0.020	0.771 ± 0.017	0.333 ± 0.009	0.194 ± 0.011	977.9
	AdaBoost [*t*_*early*_]					
5	0.732 ± 0.012	0.632 ± 0.016	0.730 ± 0.023	0.299 ± 0.008	0.248 ± 0.000	2010.5
10	0.765 ± 0.009	0.632 ± 0.014	0.793 ± 0.012	0.317 ± 0.007	0.247 ± 0.000	2977.3
15	0.768 ± 0.009	0.648 ± 0.018	0.776 ± 0.015	0.322 ± 0.008	0.247 ± 0.000	2480.3
20	0.769 ± 0.008	0.649 ± 0.021	0.777 ± 0.018	0.322 ± 0.009	0.247 ± 0.000	2685.6
36	0.769 ± 0.008	0.649 ± 0.021	0.777 ± 0.018	0.322 ± 0.009	0.247 ± 0.000	3583.5
	AdaBoost [*t*_*term*_]					
5	0.734 ± 0.012	0.637 ± 0.017	0.727 ± 0.027	0.301 ± 0.008	0.248 ± 0.000	2943.7
10	0.776 ± 0.008	0.663 ± 0.016	0.773 ± 0.013	0.330 ± 0.009	0.247 ± 0.000	2131.7
15	0.778 ± 0.008	0.666 ± 0.018	0.771 ± 0.016	0.332 ± 0.009	0.247 ± 0.000	3951.7
20	0.779 ± 0.008	0.667 ± 0.024	0.771 ± 0.021	0.332 ± 0.011	0.247 ± 0.000	3115.4
41	0.779 ± 0.007	0.669 ± 0.024	0.769 ± 0.021	0.333 ± 0.011	0.247 ± 0.000	6072.9
	Complement Naive Bayes [*t*_*early*_]					
5	0.661 ± 0.018	0.578 ± 0.028	0.663 ± 0.070	0.252 ± 0.009	0.246 ± 0.014	24.9
10	0.655 ± 0.012	0.595 ± 0.044	0.628 ± 0.079	0.249 ± 0.007	0.248 ± 0.032	30.5
15	0.660 ± 0.011	0.596 ± 0.055	0.636 ± 0.089	0.253 ± 0.007	0.247 ± 0.035	35.1
20	0.691 ± 0.009	0.621 ± 0.055	0.651 ± 0.072	0.270 ± 0.008	0.237 ± 0.034	38.2
36	0.694 ± 0.010	0.624 ± 0.055	0.653 ± 0.068	0.272 ± 0.010	0.236 ± 0.034	58.2
	Complement Naive Bayes [*t*_*term*_]					
5	0.559 ± 0.018	0.613 ± 0.042	0.464 ± 0.073	0.204 ± 0.009	0.251 ± 0.018	24.9
10	0.648 ± 0.011	0.632 ± 0.056	0.559 ± 0.092	0.246 ± 0.008	0.246 ± 0.041	32.1
15	0.654 ± 0.011	0.628 ± 0.059	0.576 ± 0.093	0.249 ± 0.008	0.246 ± 0.040	33.6
20	0.660 ± 0.011	0.635 ± 0.063	0.576 ± 0.095	0.253 ± 0.008	0.244 ± 0.042	44.8
41	0.691 ± 0.011	0.657 ± 0.061	0.600 ± 0.072	0.274 ± 0.013	0.233 ± 0.041	54.5

Based on the cross-validation results from the training cohort, the gradient-boosted trees algorithm of XGBoost was next used for subsequent validation against a large test cohort comprised of births reported by the CDC during 2014–2017. The corresponding data filtering process to arrive at the test cohort is presented in Fig 3B which indicates a cohort consisting of 10, 613, 877 births. A slightly smaller percentage of unplanned C-sections are present in the test cohort (10.1%). Guided by the classification performance of the XGBoost cross-validation results in the training dataset which considered four subsets of modeling inputs based on feature importance selection, the following modeling configurations were chosen for evaluation: 15 features from *t*_*early*_ and 20 features from the *t*_*term*_ modeling scenario. These counts were chosen as the minimum number of features for which a steady state in scoring metric performance was generally observed.

**Fig 4:**
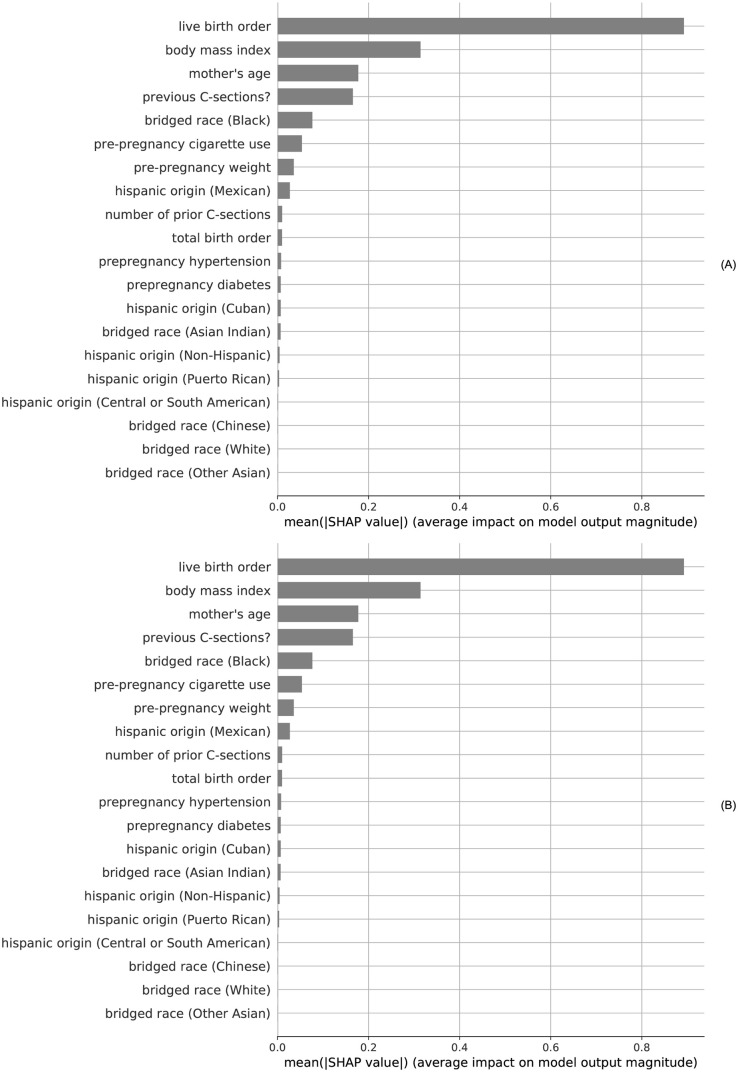
SHAP value influence of CDC inputs for the top 20 most impactful features using XGBoost on training set: (A) *t*_*early*_ scenario variables, (B) *t*_*term*_ scenario variables. The maternal modeling variables in these plots derive from CDC vital statistics data-fields and are described further in Tables 1 and 2.

The 20 most important features from the XGBoost model as identified via computation of SHapley Additive exPlanations (SHAP) values are highlighted in Fig 4 for both the *t*_*early*_ and *t*_*term*_ scenarios. In both cases, the top four most influential features are seen to be the live birth order, pre-pregnancy body mass index, mother’s age, and prior C-section indicator. In the *t*_*term*_ case, the weight gain during pregnancy is also seen to be influential as the 5^th^ most important feature.

**Fig 5:**
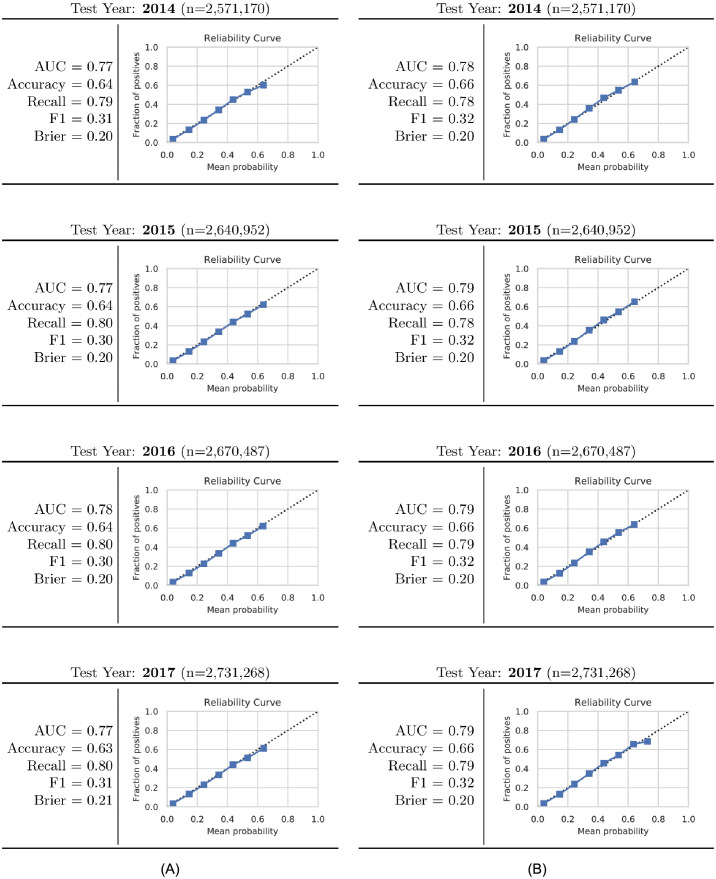
Classifier performance of XGBoost model trained using births from 2011 thru 2013 and evaluated on subsequent years 2014 thru 2017. Scoring metrics include AUC (area under the ROC curve), accuracy, recall, Brier loss, and F1 along with reliability curves to assess predictive calibration: (A) results using top 15-most influential parameters from *t*_*early*_ prediction scenario, (B) results using 20-most influential variables from *t*_*term*_.

Fig 5 presents scoring results of the trained XGBoost models applied to the test cohort on a per-year basis (four years in total) for both *t*_*early*_ and *t*_*term*_ scenarios. In addition to computing raw metrics of AUC, accuracy, recall, F1, and Brier scores, model reliability curves are presented for each year. Results are seen to be quite consistent from year to year and the reliability curves indicate good model calibration (the dashed line shown in each reliability curve provides a reference for a perfectly calibrated model). Note that raw classifier probabilities are adjusted based on the imbalance ratio which does result in a cap of maximum calibrated probabilities between 70-80%.

**Fig 6:**
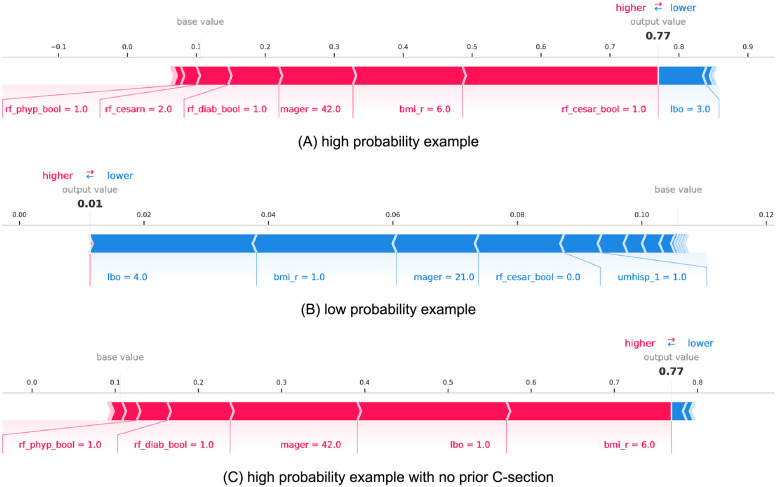
SHAP value feature influences of individual model predictions of unplanned C-section for high and low risk patients from the *t*_*early*_ prediction scenario. The maternal modeling variables in these explanation plots derive from CDC vital statistics data-fields and are described further in Tables 1 and 2. Note that per CDC encoding, a value of bmi_r = 6 corresponds to a BMI value ≥ 40 while bmi_r = 1 maps to a BMI value < 18.5. Boolean features are demarcated with 1.0 for true and 0.0 for false.

To aid in interpretation of the trained XGBoost models for individual predictions, Fig 6 presents SHAP value feature influences on the probability predictions for several test cohort samples for the *t*_*early*_ scenario. In particular, Fig 6A presents the highest probability sample ($\hat{p}=77\%$) and the 6 most influential feature values that push the probability of an unplanned C-section for this birth to be significantly higher than the mean probability value of all predictions which was $\bar{p}=10.6\%$. In addition to this mother having had multiple prior C-sections, a high body mass index (BMI ≥ 40) and pre-pregnancy diabetes and hypertension contributed to a high-probability prediction. Note that the bmi_r values recorded by the CDC correspond to six different BMI ranges delineated as follows:

1 ⇒ underweight (< 18.5)
2 ⇒ normal (18.5–24.9)
3 ⇒ overweight (25.0–29.9)
4 ⇒ obesity I (30.0–34.9)
5 ⇒ obesity II (35.0–39.9)
6 ⇒ extreme obesity III (≥ 40)

In contrast, the lowest probability sample ($\hat{p}=1.2\%$) is presented in Fig 6B highlighting the 5 most influential feature values that lowered the probability prediction of an unplanned C-section significantly below the mean. Multiple births without a prior C-section, low body mass index (BMI ≤ 18.5), and a young age are seen to lower this mother’s likelihood of an unplanned C-section. Clinically, the occurrence of one or more prior previous C-sections is understood to reduce likelihood of vaginal delivery [17]. To examine the feature influence in a birth without a prior C-section, one additional high-probability example is shown in Fig 6C. In this case, a high body mass index, pre-pregnancy hypertension and diabetes again contribute to a higher unplanned C-section probability ($\hat{p}=77\%$).

## Discussion

The current study aims to to develop a predictive model for optimal mode of delivery in pregnancy by quantify the individual risk of having an unplanned C-section following an attempted vaginal delivery. Clinically, the motivation for such a predictive tool is to aid in the decision making process to offer and consider an elective C-section for patients identified with a higher risk of having an unplanned C-section. A benefit and motivation for choosing to use national vital statistics data from the CDC is that it includes a comprehensive, nearly complete sample of *all* U.S. births over multiple years. This dataset thus contains ample samples for training and evaluation of multiple models over a large target population and development of the models in this context minimizes the risk of overfitting while maximizing the probability of capturing individual variation.

Current conventions regarding mode of delivery promote vaginal delivery in most patients. In part, this is motivated by the fact that lower rates of maternal mortality and morbidity are associated with vaginal delivery when compared to all C-section deliveries (both planned and unplanned). Indeed, this sentiment is reflected in current guidelines regarding C-section on maternal request [18, 19]. However, planned versus unplanned C-sections have different risk profiles and approximately one in ten women attempting vaginal birth ultimately delivers via an unplanned C-section during labor due to maternal or fetal indications. These unplanned C-sections are not only much riskier for both the mother and the baby when compared to vaginal delivery, but also when compared to an electively planned C-section before labor. In particular, an unplanned C-section in labor is associated with a twofold higher risk of maternal mortality and morbidity, and a two- to fivefold higher rate of perinatal mortality and morbidity versus an electively planned C-section [2–7, 20]. Thus, there is a clear need to individualize the decision regarding mode of delivery. For those women who are at high risk of having an unplanned C-section in labor, a planned C-section before onset of labor is likely the much safer option.

More detailed clinical guidance on choosing the mode of delivery has focused on patients who have had a previous C-section. For example, an analysis of ∼12K women across nineteen academic medical centers was used previously to develop a calculator predicting probability of a successful vaginal birth after Cesarean delivery (VBAC) [21]. In contrast, the current effort considers all labored births during model development and testing and leverages significantly larger cohorts using CDC data for model development and, more importantly, model validation (with *n* ≈ 6.5M births for training and *n* ≈ 10.6M births for testing in the current work).

A more recent machine learning approach was used for the prediction of successful vaginal delivery [22]. This publication used information available at admission to the labor and delivery unit and during the first stage of labor to predict occurrence of vaginal delivery. Thus, this study addresses a different clinical situation and one that precludes or limits the decision options before labor onset, such as elective cesarean delivery. Moreover, this study was done in a sample of the target population from a single center limiting the generalizability of the study findings, further limited by a low rate of unplanned cesarean deliveries, approximately half of that observed in the US. Furthermore, the resulting calibration of internal validation results showed substantial overestimation of risk. In contrast, the calibration results demonstrated in the current effort were very good over a large range of risks which is critical for decision making contexts.

Cross-validation results (Table 3 obtained from the best of three different machine-learning classification algorithms considered herein showed AUC and recall scores of 77% and 79% respectively for the *t*_*early*_ prediction scenario using 15 modeling features. Similar results for the *t*_*term*_ prediction scenario were observed with AUC and recall scores of 78% and 77% respectively with 20 modeling features employed. Exercising the final XGBoost model trained against births from 2011–2013 across multiple external validation years from 2014–2017 yielded nearly identical AUC and recall scores as the original k-fold cross-validation. In particular, AUC scores fell between 77–79% for both *t*_*early*_ and *t*_*term*_ while recall scores were in the range 78–79%. The results were also very consistent year to year from 2014–2017. After accounting for classification imbalance, the resulting models are also seen to have excellent calibration properties when evaluated against the test cohort. Good calibration performance is an important requirement for any potential clinical model as it reflects the degree to which a model’s predicted probability estimates the true correctness likelihood [23].

Based on these results, we conclude that there is indeed sufficient information available in national vital statistics from extended birth-certificate data for effective prediction of the optimal mode of delivery. In the analysis, a maximum of 41 features were considered after one-hot encoding categorical variables for the *t*_*term*_ prediction scenario (36 features for the *t*_*early*_ scenario). For the boosted gradient-tree method of XGBoost, we computed SHapley Additive exPlanations (SHAP) values [24] to calculate feature importance based on the average impact on model output for the top 20 features presented in Fig 4. Similar to existing models for VBAC prediction, a mother’s age and starting body mass index are seen as important predictive features in the current models. In addition to live/total birth order and number of previous C-sections, other variables from the *t*_*early*_ scenario making the top 10 most influential features include mbrace_2 (indicating race = Black), cig_0 (pre-pregnancy smoking), pwgt_r (pre-pregnancy weight), and umhisp_1 (indicating a Mexican hispanic origin). Three additional features available near the end of pregnancy are seen to be in the top 10 for average model impact in the *t*_*term*_ prediction scenario, namely wtgain_rec (weight gain), combgest (gestational age), and previs_rec (number of prenatal visits).

In summary, this work has developed an individualized predictive model for optimal mode of delivery creating a clinically useful aid in decision making regarding the safest mode of delivery and demonstrated the usefulness of national vital statistics combined with machine-learning techniques for this type of analysis. Several classifiers were considered with the gradient-boosted machine variant from XGBoost chosen as the best performer. A particular strength of this model lies in aggregating the predictive power of a large number of risk factor combinations and protective characteristics rather than relying solely on a handful of features that are observed to be influential on average. Furthermore, the large combination of risk factors and protective characteristics provide excellent predictive accuracy as evidenced by the well-calibrated results obtained during external validation across multiple test years. The resulting model is dual-pronged targeting usage scenarios at the beginning of pregnancy (*t*_*early*_) and near the time of labor and delivery (*t*_*term*_) allowing for individualized risk prediction and feedback at two points during pregnancy. Furthermore, the model is amenable for implementation into clinical practice via an interactive front-end to assess individualized risk and weigh the influence associated with different risk factors before and during pregnancy.

## Methods

For this work, the analysis toolchain uses a custom Python 3.x application that was developed to first parse and load raw datafiles as published by the CDC on a year by year basis into Pandas arrays [25]. CDC birth files published by the CDC have companion user guides which detail available variables each year along with their physical location (columns) within the flat ASCII datafile. Unfortunately, the location of variables of interest are not necessarily consistent from year to year and one must be careful to account for these subtle changes. Furthermore, many variables also have separate reporting flags which are used to indicate whether the birth is reported using the revised (2003) certificate of live birth [26]. These reporting flags must be queried for relevant variables of interest to confirm data availability during parsing. To allow for a flexible runtime description of CDC variables and reporting flags, the analysis pipeline code developed herein utilizes an INI style input file to document variable locations on a per-year basis. The following highlights one example stanza of the input description for the bmi_r variable that is included in the *t*_*early*_ prediction scenario described in Table 1.

[cdc/varindex/bmi_r]

len = 1

type = int

2011 = 533

2012 = 533

2013 = 533

2014 = 287

2015 = 287

2016 = 287

2017 = 287

2011_flag = 576

2012_flag = 576

2013_flag = 576

2014_flag = 282

2015_flag = 282

2016_flag = 282

2017_flag = 282

The syntax above provides information on the location of bmi_r indicating an integer field length of one which is located in column number 533 for years 2011–2013. However, beginning in 2014, the location changes to column number 287 and remains there through 2017. Similar information is provided for the location of a reporting flag for this variable which also changes field location in 2014. Using this flexible input parsing description for all parameters of interest, we were able to accommodate yearly changes to underlying CDC file formats to ultimately assemble a large aggregate dataset from 2011–2017 consisting of over 25M births that were reported using the revised birth certificate format.

One additional subtlety that arose during CDC data parsing concerns the consistent availability and definition of mother’s bridged race during the reporting years 2011-2017. In particular, the allowable values for the mbrace variable reduced significantly in 2014 to include only 4 race identifier values versus the 18 identifiers defined previously from 2011–2013. Fortunately, additional race recode variables were also introduced in 2014 and we identified one new variant (mrace15) with sufficient overlap with the original variable. The race identification values for this new variable are identical to the previous mbrace values with the exception of how bridged multiple races are identified. With mrace15, a single categorization is used to identify multiple race while the original mbrace variable delineated a mother’s bridged race into four variants. To derive a consistent race designation for all analysis years considered herein, we thus collapsed the bridged multiple race options present in years 2011–2014 into a single marker in combination with the use of mrace15 for later years.

Once the relevant CDC data has been parsed and loaded, the starting analysis cohort is assembled using the logic identified in Fig 2. Further filtering is applied to restrict analysis to singleton births with labor attempted and cephalic presentations. Births in which modeling variables from Table 1 are missing are also dropped from the analysis and specific data counts for each step of the filtering process are included in Fig 3. Note that of the records dropped due to missing data, wtgain_rec was one of the larger contributors with 4.2% of eligible births dropped from the training cohort and 3.5% from the test cohort. The smoking indicators (cig_n) also had a larger contribution to missing data in the training cohort with 4.8% of eligible births being dropped. This reporting prevalence of cig_n is improved significantly for latter years with only 0.6% of eligible births dropped in the test cohort. After filtering, two additional data transformations are applied to prepare for subsequent classification training and evaluation. First, the two categorical features highlighted in Table 1 are one-hot encoded which increases the modeling state space to a maximum of 41 features. Second, six *yes/no* textual risk factors parsed from raw CDC data are converted to binary counterparts; these are applied to rf_ppterm, rf_cesar, rf_diab, rf_phyp, rf_ghyp, and rf_gest with a _bool suffix appended to the variable names.

From a machine learning perspective, the mode of delivery analysis is poised as a supervised learning problem with a binary output class that corresponds to whether a birth was delivered vaginally (*class* = 0) or via an unplanned C-section (*class* = 1). As is the case frequently encountered in medicine, the distribution for the output class is unbalanced with 10.3% of the data samples observed in the positive class in CDC data from 2011–2017. To test applicability of classification techniques, three different algorithms are trained and evaluated via cross-validation using the training cohort. These include AdaBoost [27], a meta-estimator which combines weighted predictions from a sequence of weak learners; XGBoost [28], an optimized gradient boosting library; and Complement Naive Bayes [29], an updated variant of the classic Naive Bayes classifier that is better suited for imbalanced datasets. In all cases, we leverage the scikit-learn [30] interface to these algorithms for training, cross-validation, scoring evaluation, and prediction.

Given the imbalance present in our unplanned C-section classification variable, additional care must be taken to appropriately weight samples during the training phase and we leverage scikit’s compute_sample_weight function in “balanced” mode to compute individual sample weights that are inversely proportional to class frequency. These weights are then provided as input to each of the underlying classifiers fit() method. We chose this approach over under sampling [31] to avoid ignoring the majority of samples (births) available during training.

Feature selection results are also evaluated in the context of cross-validation using the training set and classification performance was computed for the top 5, 10, 15, and 20 subfeatures in terms of importance for both *t*_*early*_ and *t*_*term*_ scenarios (Table 3). To choose the subfeatures, each of the three machine-learning algorithms considered were first trained against the entire training set with all available features. Then, feature importance for each input was computed in one of two ways depending on the classifier. For XGBoost, the feature order was determined using mean SHAP value [24] impacts on model output. For the other two classifiers, the feature_importances method was exploited in scikit-learn which orders model features using gini importance [32]. Subsets of the most-important features were then used in ten-fold cross-validation to assess model performance with increased feature counts for each algorithm. Note that the parallel (threaded) capability of XGBoost was exploited on 44 cores to reduce execution time. The other classifiers do not have a parallel implementation within scikit-learn and were executed serially. Based on the results obtained with cross-validation, we identified XGBoost as the best performer for the algorithms considered and chose model configurations of 15 features for *t*_*early*_ and 20 features for *t*_*term*_ for subsequent evaluation against the test cohort (as no improvement in scoring metrics was observed with additional features added).

Validation results presented in Fig 5 are obtained using XGBoost with chosen subfeature counts by training against *n* = 6,530,467 samples from the training cohort (births during 2011–2013) and testing against *n* = 10,613,877 samples from the test cohort (births during 2014–2017). Five scoring metrics are computed against yearly subsets of the test cohort using standard classification scoring routines provided by the sklearn.metrics class. Predicted probabilities for the test samples are computed using each classifiers predict_proba method. Reliability (model calibration) curves are generated by computing histograms of predicted model probabilities into a maximum of 10 bins and comparing the mean probability within each bin to the fraction of true positives from samples within the bin [33]. Note that while the maximum bin count considered is 10, we restrict the highest bin to have a minimum of at least 100 samples.

When evaluating model calibration, additional treatment is necessary to adjust resulting classifier probability outputs to account for the imbalanced prevalence of unplanned C-sections. In this case, we assume a similar prior between training and test populations and use the imbalance ratio observed from the training cohort ($\beta =\frac{700,943}{5,829,524}=12.02\%$) to adjust raw classifier probabilities (*p*) to a calibrated probability (*p*′) as follows [34]:

$$\begin{array}{c} p^{\prime }=\frac{\beta p}{\beta p-p+1} \end{array}$$ (1)

The updated probability distributions obtained using this calculation are then used to generate the reliability curves that are included in Fig 5. Note that the transformation applied via Eq 1 does limit the maximum possible classifier probabilities and consequently, the largest mean probability seen in the reliability curves is ∼73%.

To interrogate feature influence of trained XGBoost models for individual birth predictions, we leverage the force_plot utility provided by the SHAP library [35]. Three examples using this approach (two with high probability and one with low) are shown in Fig 6, although similar plots can be generated for any prediction to help aid in explaining which maternal characteristics lead to an individual patient’s probability prediction.

Note that the raw data utilized to complete this analysis is available for public download from the CDC’s Vital Statistics online portal. Relevant live-birth data is obtained from the denominator files included in period linked birth-infant death data files from 2005 to 2017. The uncompressed size of these files totals approximately 50GB.

The companion Python utilities and Jupyter notebooks used to complete this analysis are available via GitHub [36]. All analysis was completed using the developed source-code files starting from raw CDC birth files on computational resources housed at the Texas Advanced Computing Center (TACC). The data analysis environment was containerized using Docker [37] and executed within a Linux HPC cluster running OpenHPC [38] with 64GB of ram/node. Note that a self-contained Dockerfile that defines the Python analysis platform with all required computing modules and Jupyter support is also included within the GitHub repository.
